# Understanding User Perspectives to Inform Personalized Physical Activity Promotion in a Health Care App: Qualitative Focus Group Interview Study

**DOI:** 10.2196/85390

**Published:** 2026-03-09

**Authors:** Yutong Shi, Jihoon Kim, Ryoko Mizushima, Shinichiro Mizuno, Tomoko Yanagisawa, Yoshio Nakata

**Affiliations:** 1 Institute of Health and Sport Sciences, University of Tsukuba, 1-1-1 Tennodai, Tsukuba, 305-8574, Japan, 81 29-853-3957; 2 Department of Epidemiology and Biostatistics, National Institute of Public Health, Wako, Japan; 3 Solution Department, Wellmira Inc, Tokyo, Japan

**Keywords:** physical activity promotion, health care app, personalization, focus group interview, qualitative research, social ecological model, behavioral motivators, physical activity barriers, digital health

## Abstract

**Background:**

Health care apps are widely used to support weight loss and lifestyle modification. Many of these apps offer tailored feedback on dietary intake and nutritional behavior. However, most lack personalized features that promote physical activity (PA), which is important for weight management, metabolic health, and chronic disease prevention. To develop future personalized PA promotion functions, it is essential to understand users’ perceptions of PA.

**Objective:**

This study aimed to explore health care app users’ perception of PA, including perceived motivators and barriers.

**Methods:**

A qualitative study was conducted using focus group interviews with health care app users. Participants were recruited regardless of age, sex, or body mass index. A thematic analysis was conducted using a combination of inductive and deductive approaches. Question 1 (“How do you perceive the importance of physical activity?”) was analyzed inductively, whereas questions 2 (“What are the motivating factors for engaging in physical activity?”) and 3 (“What are the barriers to engaging in physical activity?”) were analyzed deductively based on the social ecological model.

**Results:**

Eleven participants were interviewed and were unfamiliar with the term “physical activity” but recognized the importance of movement and reducing sedentary behavior. The identified motivators included improvements in mood; changes in physical appearance; support from family; alignment with personal routines and conditions (eg, goal setting, feedback, reminders, and praise); and tailoring to physical condition, daily schedules, and weather. The reported barriers included time restrictions due to work, fatigue, weather, remote work, and social pressure in workplace settings.

**Conclusions:**

This study provides user-informed insights that can inform the design of personalized approaches better aligned with daily routines, competing demands, and situational barriers. Future work should evaluate how incorporating such user perspectives into personalized support strategies affects engagement and PA.

## Introduction

### Background

Physical inactivity (PA) is globally recognized as a critical public health issue that significantly contributes to the prevalence of obesity, cardiovascular diseases, type 2 diabetes, and other chronic conditions. The World Health Organization emphasizes that PA is essential for preventing and managing chronic diseases among diverse populations [1]. To achieve these health benefits, the World Health Organization recommends that adults aged 18 to 64 years should engage in at least 150 to 300 minutes of moderate-intensity aerobic PA or 75 to 150 minutes of vigorous intensity PA weekly [2]. Despite robust evidence supporting the benefits of PA, many individuals fail to achieve recommended levels due to perceived barriers, such as lack of time, fatigue, and environmental constraints (such as limited space and pollution) [3].

The recent development of mobile health apps has provided promising tools for behavioral modification, offering convenient and cost-effective health care interventions [4]. Advances in smartphone technology and wearable devices have enabled the integration of numerous basic personalized features, such as goal setting, activity reminders, self-monitoring dashboards, and adaptive feedback functions [5,6]. Recently, artificial intelligence–driven algorithms have been developed to deliver contextually tailored content that considers user real-time behavior, environmental conditions, and individual preferences [7].

Personalization generally refers to adapting intervention content, timing, or delivery to individual characteristics, preferences, and circumstances [8]. In developing personalized app functions, it is recommended to consider user factors, such as sociodemographic characteristics, adaptive goals, and timely feedback [9]. However, commercial PA apps often lack proactive and context-aware features (eg, tailored plans and context-aware prompts), and this limited ability to tailor support to individual differences and diverse circumstances may undermine effective and sustained user engagement [10].

User-centered design and qualitative inquiry are well-established approaches in digital health research for exploring users’ needs and experiences. To enhance the appropriateness and effectiveness of personalization in digital health interventions, it is critical to understand user perspectives and needs rather than relying on developer assumptions only [11]. Qualitative methods are particularly well suited for the detailed exploration of subjective experiences, preferences, and perceptions, and focus group interviews (FGIs) are a widely used qualitative approach for generating data through group discussion [12,13].

### Objectives

Therefore, this qualitative study aimed to explore users’ experiences and preferences related to PA promotion using FGIs. The findings of this study will contribute valuable knowledge for the development of more engaging, personalized, and context-sensitive digital health interventions.

## Methods

### Study Design

This study used a qualitative research design utilizing FGIs and was reported in line with the COREQ (Consolidated Criteria for Reporting Qualitative Research) checklist (Checklist 1) [14].

### Participants and Recruitment

Participants were broadly recruited through a health care app, CALOmama PLUS (Wellmira Inc), with no restrictions on sex, age, or BMI. CALOmama PLUS is a health care app with which users can register their daily information (diet, exercise, etc), and artificial intelligence instantly provides advice based on the registered information [15]. Interviews were conducted either face-to-face or online. In both settings, participants received a detailed explanation of the study via email in advance and were asked to provide preliminary consent to indicate their availability. The purpose and content of the study were explained again before the interview.

### Data Collection and Survey

FGIs were conducted face-to-face and online via Google Meet (Google LLC). Based on participants’ availability, three 1-hour interviews were held with 5, 2, and 4 participants (N=11). Focus groups are typically kept small to facilitate interaction and ensure that each participant has sufficient opportunity to contribute, with an “ideal” group size of approximately 4 to 8 participants suggested [12]. Interviews were recorded using digital voice recorders (ICD-TX650; Sony) and subsequently transcribed. All FGIs were facilitated by YN, a professor with extensive experience in conducting interviews. In addition, 4 researchers (YS, RM, SM, and TY) also participated. Two researchers (YS and RM) acted as co-facilitators, supporting the lead facilitator by asking clarification and follow-up questions to deepen participants’ responses and monitoring group dynamics to ensure balanced participation. Two other authors (SM and TY) are employees of the health care app company. For participants attending face-to-face sessions, interviews were held at the company site for logistical reasons, with SM and TY present to support the on-site operation. Their involvement in the discussion was limited to a brief, clearly separated end-of-session segment addressing general app-use impressions (eg, usability). These ancillary questions were not part of the semistructured interview guide, and the data from this segment were excluded from the thematic analysis, as they were outside the study’s primary aim. To minimize potential bias related to company affiliation, the core focus group discussions were led by academic researchers, and thematic coding and interpretation were conducted by researchers independent of the company’s development team.

The semistructured focus group guide was created by YS and reviewed by all authors to reduce assumptions and potential bias of the first author. The review specifically focused on (1) ensuring alignment with the study aims, (2) reducing leading or value-laden wording, (3) improving clarity and neutrality of probes, and (4) confirming feasibility for a group interview. In addition, coauthors with prior qualitative interviewing experience (including JK, YS, and RM) conducted a targeted content and face-validity review, and revisions were made through iterative discussion among the research team before data collection. The semistructured FGI guide is available in Multimedia Appendix 1.

The questionnaire, sent in advance by email, collected information on 3 domains: baseline characteristics, health care app usage, and PA levels. Baseline characteristics included sex, age, height, weight, smoking status, exercise habits, walking habits, employment status, educational background, household income, cohabitation status, and marital status. For health care app usage, participants were asked about the duration of usage, reasons for use, and perceived physical changes after use. PA was assessed using the Global Physical Activity Questionnaire [16]. The interview questions were as follows: (1) How do you perceive the importance of PA? (2) What are the motivating factors for engaging in PA? (3) What are the barriers to engaging in PA?

### Data Analysis

All FGI sessions were audio-recorded and transcribed. The transcripts were organized and managed using the NVivo software (MacOS Release 14.23.4; Lumivero) to facilitate thematic analysis. Thematic analysis is a qualitative method for identifying, analyzing, and reporting themes within data relevant to research questions [17]. Themes may be derived inductively from the data or deductively based on existing theories or frameworks [18]. Researchers are encouraged to use either or both approaches depending on the research purpose [19].

In this study, an inductive approach was applied to analyze responses to question 1, whereas a deductive approach based on the socioecological model was used for questions 2 and 3 [20]. This model considers not only individual factors but also interpersonal, organizational, and community-level environmental factors. Two researchers (YS and JK) independently reviewed the transcripts, categorizing content into “factors,” “categories,” and “codes.” Discrepancies were discussed and resolved in consultation with a third researcher (YN) until consensus was achieved. The company-employed authors (SM and TY) did not participate in coding or in decisions regarding theme definition.

All interviews were conducted in Japanese, and the first author translated selected illustrative quotations into English. Translations were carefully checked against the original Japanese transcripts to preserve semantic meaning. Ambiguous phrasing was refined through discussion among the authors with reference to the original text.

### Ethical Considerations

The study protocol was reviewed and approved by the Institute of Health and Sports Sciences at the University of Tsukuba (approval number: Tai 023‐140). Before the FGI, the researcher (YN) introduced the research staff and explained the study purpose and procedures. Written and verbal informed consent was obtained from face-to-face and online participants, respectively. To protect participants’ privacy and confidentiality, all collected data were deidentified and stored on password-protected devices with access restricted to the principal investigator and research team. Any documents containing identifiable information were kept in a locked cabinet/locker in the research office, and only aggregated results are reported; quotations were presented without information that could identify individual participants. Participants received a QUO card (JPY 3000; US $19.18) as compensation for their time.

## Results

### Participant Characteristics

Eleven app users (3 men and 8 women) participated in the FGI; their characteristics are listed in Table 1. Participant ages ranged from 31 to 76 years. Of the participants, 2 lived alone, whereas 8 were married. Over half (6 participants) had been using the app for less than 1 year. The reasons for use included dietary (5 participants), weight (4 participants), and general health (2 participants) management. The range of values for BMI, occupational moderate-to-vigorous physical activity (MVPA), transport-related MVPA, leisure-time MVPA, and sitting time were 17.9‐28.6 kg/m^2^, 0‐180 minute/week, 0-840 minute/week, 0‐560 minute/week, and 180‐840 minute/day, respectively.

**Table 1.** Demographic characteristics of the participants (N=11).

Variables	Participants										
	A	B	C	D	E	F	G	H	I	J	K
Sex	Male	Female	Male	Female	Female	Female	Female	Female	Female	Male	Female
Age (y)	76	37	70	53	49	31	55	44	55	65	33
BMI (kg/m^2^)	19.79	24.03	17.93	19.81	24.52	19.47	21.36	23.63	28.57	24.39	22.03
Exercise habits	Yes	No	Yes	No	No	Yes	Yes	No	Yes	Yes	No
Walking habits	Yes	No	Yes	No	No	Yes	Yes	No	Yes	Yes	No
Educational level	UG^a^	UG	UG	UG	JC^b^ or TCG^c^	UG	JC/TCG	UG	UG	UG	JC/TCG
Duration of app use (y)	3‐5	<1	≥5	1‐3	<1	1‐3	<1	<1	<1	3‐5	<1
Reason for starting app use	Weight management	Diet management	Diet management	Health management	Diet management	Diet management	Weight management	Health management	Diet management	Weight management	Weight management
Occupational MVPA^d^ (min/wk)	0	0	0	0	0	0	0	0	180	0	0
Transport-related MVPA (min/wk)	0	150	0	360	0	80	840	60	120	150	450
Leisure-time MVPA (min/wk)	180	240	560	360	120	240	0	30	0	180	150
Sitting time (min/d)	720	600	720	330	360	480	840	480	240	180	720

a UG: undergraduate degree.

b JC: junior college degree.

c TCG: technical college degree.

d MVPA: moderate-to-vigorous physical activity.

### Question 1 Analysis

#### Overview

The responses to question 1 were analyzed using an inductive approach, and the FGI content was broadly categorized into “Awareness” and “Consciousness.” To understand the current perceptions of PA, awareness was subcategorized into “Status.” Furthermore, subcategories were defined as “Exercise” and “Daily activities” under “Consciousness.” All participants were aware of the negative effects of prolonged sedentary behavior, whereas the term “physical activity” was not widely recognized. Some noted spending long periods sitting both at home and at work and observed a reduction in step counts due to working at home (Table 2).

**Table 2.** Summary of awareness of the importance of physical activity (question 1).

Categories and subcategories	Codes
Awareness	
Status	Lack of awareness
Consciousness	
Exercise	Importance
Daily activities	Household activities Commuting activities Work activities

#### Awareness

Recognition of the importance of PA was classified under the subcategory “Status” and coded as “Lack of awareness.”

*This is the first time I’ve heard the term “physical activity,” and I was not clear on the difference from exercise.*

[Participant B; Lack of awareness]

*Aside from intentional exercise, I don’t really engage in the kind of physical activity defined today. I do some gardening, but I don’t think it counts for much.*

[Participant C; Lack of awareness]

*I just learned about physical activity today, so I haven’t really paid attention to my daily movements. But now I realize that even small efforts can be effective.*

[Participant B; Lack of Awareness]

#### Consciousness

Within “Consciousness,” 2 subcategories were identified: “Exercise” and “Daily activities.” Many participants expressed an awareness of the importance of exercise; therefore, the subcategory “Exercise” was coded as “Importance.” For “Daily activities,” based on the Global Physical Activity Questionnaire domains and participant statements, codes were created for “Household activities,” “Commuting activities,” and “Work activities.”

*I think exercise is important and try to do it as much as possible. I’ve been swimming since I was a child and still swim during my free time on weekends.*

[Participant B; Importance]

*I want to move as much as possible because it helps with weight management and posture and reduces stress.*

[Participant F; Importance]

*I make a conscious effort to walk around the house and go shopping to avoid being sedentary.*

[Participant E; Household activities]

*Even if I’m doing housework, I notice a big difference in calories burned and steps taken between days spent entirely at home and days when I go out, so I try to go out at least once a day.*

[Participant K; Commuting activities]

*During commutes, I try not to sit on the train and use the stairs instead of escalators.*

[Participant K; Commuting activities]

*Remote work has reduced both my physical activity and exercise, making me realize I need to move more.*

[Participant B; Work activities]

*I try to stand up and move my legs even when working at my desk.*

[Participant A; Work activities]

### Question 2 Analysis

#### Overview

A deductive approach was used for the second FGI question, which explored factors promoting PA. The content was classified using the social ecological model [16] into “Individual factors,” “Sociocultural environmental factors,” “Physical environmental factors,” and “Organizational factors.” In summary, individual factors, such as health checkup results and mood, were frequently cited as triggers for increasing PA. Family and friends were considered important for promoting PA, and support from health care providers was considered essential. Other factors included the work environment and mandatory workplace instructions (Table 3).

**Table 3.** Summary of motivating factors for promoting physical activity (question 2).

Factors and categories	Codes
Individual factors	
Demographic	Appearance
Biological	Biological health
Psychological	Mood Experience
Sociocultural environmental factors	
Social networks	Family Peers Social media
Smartphone application	Goals and planning Feedback and monitoring Reward
Physical environmental factors	
Built environment	Office ergonomics
Organizational factors	
Policy	Directives

#### Individual Factors

Individual factors were classified as demographic, biological, and psychological. Demographic and biological categories were coded as appearance and biological health, respectively. Psychological factors were subdivided into codes, including mood and experience.

*Seeing physical changes, such as a slimmer body line or fitting into previously tight clothes, motivates me to keep going.*

[Participant E; Appearance]

*Health checkup results are a major trigger for me to start exercising.*

[Participant C; Biological health]

*Enjoyable and fun exercise sessions, especially with others, make it easier to continue.*

[Participant F; Mood]

*Exercising helps clear my mind and improves my mood, which motivates me to continue.*

[Participant F; Mood]

*Past experiences of successfully losing weight through exercise and diet encourage me to try again.*

[Participant E; Experience]

#### Sociocultural Environmental Factors

We classified sociocultural environmental factors into social networks and smartphone apps. In addition, the social network category was subdivided into codes, including family, peers, and social media. The smartphone app was subdivided into codes, such as goals and planning, feedback and monitoring, and reward.

*Having family or friends to exercise with makes it easier to stay motivated.*

[Participant E; Family]

*Swimming classes with friends motivate me to continue exercising regularly.*

[Participant B; Peers]

*I often walk or attend gym sessions with friends nearby, which helps sustain physical activity.*

[Participant F; Peers]

*Watching YouTube videos or seeing others’ experiences on social media inspires me to be more active.*

[Participant E; Social media]

*If an app clearly shows how to achieve weight loss through recommended routines, I would be motivated.*

[Participant H; Goals and planning]

*Setting clear goals and plans in the app, receiving feedback and monitoring, and getting encouragement or rewards are all motivating.*

[Participant I; Feedback and monitoring]

*Immediate positive reinforcement from the app for even small activities would be encouraging.*

[Participant A; Reward]

*Receiving compliments from characters on the application motivates me.*

[Participant B; Reward]

#### Physical Environmental Factors

We classified physical environmental factors into built environments and coded the category as office ergonomics.

*I wish my company had a treadmill desk so I could work while walking.*

[Participant B; Office ergonomics]

#### Organizational Factors

We classified organizational factors into policies and coded them as directives.

*Mandatory standing breaks before meetings would be very beneficial.*

[Participant F; Directives]

*Forced participation, like mandatory office attendance or children’s school events, increases my physical activity more than voluntary measures.*

[Participant H; Directives]

*Notifications on computers for breaks would be helpful.*

[Participant F; Directives]

### Question 3 Analysis

#### Overview

A deductive approach was also used for the third FGI question, which explored barriers to PA, and the content was classified using the social ecological model into “Individual factors,” “Sociocultural environmental factors,” and “Physical environmental factors.” In summary, the most frequently cited barriers were time constraints due to work-life balance and weather conditions. Many participants noted that remote work increased their sedentary time and made it more difficult for them to be physically active. Other barriers included a lack of knowledge about the benefits of PA and concerns about being watched by others at work (Table 4).

**Table 4.** Summary of barriers to promoting physical activity (question 3).

Factors and categories	Codes
Individual factors	
Demographic	Age Socioeconomic status
Biological	Biological health Fatigue
Psychological	Habit Attitude Knowledge
Work-life balance	Restriction
Sociocultural environmental factors	
Social networks	Colleagues
Physical environmental factors	
Natural environment	Weather
Work environment	Work from home Facility

#### Individual Factors

We classified individual factors into demographic, biological, and psychological factors, as well as work-life balance. Demographic and biological categories were subdivided into codes, such as age, socioeconomic status, biological health, and fatigue. Psychological factors were subdivided into codes, including habits, attitudes, and knowledge. Work-life balance was coded as restrictions.

*Due to my age, I rarely go out except when necessary, and even then, I usually drive rather than walk.*

[Participant C; Age]

*Financial constraints make it difficult to continue going to a sports club.*

[Participant E; Socioeconomic status]

*Injuries or health issues make it hard to be active.*

[Participant C; Biological health]

*Fatigue after work is a major barrier.*

[Participant E; Fatigue]

*Convenient services like home delivery reduce the need for movement.*

[Participant H; Habit]

*I tend to relax on the sofa and use my smartphone during free time.*

[Participant K; Habit]

*I’m not sure if small activities outside of intentional exercise are really beneficial.*

[Participant C; Knowledge]

*Time constraints due to work and household responsibilities make it difficult to be active.*

[Participant H; Restriction]

#### Sociocultural Environmental Factors

Sociocultural environmental factors were classified as social networks. In addition, the social network category was coded as colleagues.

*It’s hard to move when others are around at work.*

[Participant F; Colleagues]

#### Physical Environmental Factors

We classified physical environmental factors into natural and work environments. The natural environment category was coded as weather categories. The work environment was subdivided into codes, including work from home and facilities.

*Hot or rainy weather makes it difficult to exercise outdoors.*

[Participant A; Weather]

Menstrual periods and rainy days reduce my desire to go out. Low atmospheric pressure, causing headaches, is also a barrier.

[Participant F; Weather]

*Working from home leads to long sedentary periods and reduced physical activity.*

[Participant B; Work from home]

*During telework, sitting all day at home, I often realize I’ve only taken about 400 steps, which is unhealthy.*

[Participant H; Work from home]

*On weekdays, commuting was my primary physical activity. With partial teleworking recently, physical activity drastically decreased.*

[Participant B; Work from home]

*My previous job involved sitting at a desk reading and writing, with little physical activity. Even commuting was by car.*

[Participant C; Facility]

## Discussion

### Principal Findings

In this study, FGIs were conducted to explore users’ understanding of PA and their perceived motivators and barriers in promoting PA in daily life. Question 1 assessed participants’ perceptions of the importance of PA, whereas questions 2 and 3 examined motivating factors and barriers.

Regarding question 1, although participants had limited awareness of the term “physical activity,” they demonstrated an understanding of exercise and the need to move their bodies. Similarly, a previous study [21] reported low awareness of Japan’s PA guidelines among 7000 adults aged 20 to 69 years registered with an online survey company, with 1.7% and 5.3%‐13.4% demonstrating spontaneous and assisted recalls, respectively. These findings indicate that public awareness and knowledge of PA guidelines remain limited, underscoring the need for increasing public awareness and understanding of PA guidelines.

Conversely, as indicated by participant demographic characteristics, over half (6 of the 11 participants) reported regular exercise habits. According to the 2023 National Health and Nutrition Survey [22], 36.2% of men and 28.6% of women have regular exercise habits, showing no significant changes over the past decade. However, compared with 2019 (men: 33.4%, women: 25.1%), a slight increase was observed. Even among participants without regular exercise habits, comments indicated conscious efforts to reduce sitting time and engage in incidental PA. Given participants’ desire for reminder functions in apps, implementing education and promotion strategies using health care apps could be beneficial.

In question 2, motivating factors for promoting PA were analyzed using the social ecological model. At the individual level, improvements in appearance, medical examination results, and enhanced mood were identified as motivators. These findings align with previous research; for instance, a previous study [23] reported health concerns, appearance satisfaction, and emotional encouragement as motivators for individuals with obesity attempting weight loss. Moreover, positive mood after exercise has been reported as a key motivator for PA promotion, significantly correlating with achieving recommended levels of leisure-time walking [24].

At the sociocultural level, social networks and smartphone apps were identified as important factors. Participants highlighted family and peer support as crucial motivators. Similarly, one previous research [25] has emphasized that spousal support significantly influences exercise adherence in couples with diabetes. Another study [26] further demonstrated that changes in PA were positively correlated between spouses, indicating the importance of targeting couples in interventions. Participants also emphasized the motivational role of social media, consistent with prior studies showing increased exercise intention following exposure to fitness-related content [27].

The key motivating functions of smartphone apps included goal setting, feedback and monitoring, and rewards. A previous study [28] has similarly emphasized the importance of goal-setting compliance during the initial weeks of weight-loss interventions. Behavioral planning using smartphone apps has shown moderately positive associations with increased PA in individuals with cardiovascular diseases [29]. Furthermore, personalized feedback tailored to user preferences significantly enhanced PA and weight loss outcomes [30]. Digital self-monitoring techniques have also demonstrated significant effects on weight loss and moderate PA promotion in overweight adults [31]. Moreover, rewards and behavioral planning are effective strategies for promoting long-term PA [32]. Incorporating these behavioral change techniques into health care apps is expected to enhance the future promotion of PA.

Physical, environmental, and organizational factors primarily focus on workplace issues. Participants highlighted the need for improved workplace environments and policies, particularly considering modern sedentary work practices. This finding aligns with that of prior studies, suggesting that workplace interventions effectively reduce sitting time and promote PA [33]. Therefore, developing feasible intervention programs using health care apps that address these factors is essential.

In question 3, barriers to PA were analyzed using the social ecological model. Individual factors prominently emerged, with biological health issues, such as pain and injuries, identified as significant barriers, consistent with previous findings [34-37]. Fatigue and socioeconomic factors were also mentioned, aligning with existing research. Interviews with African American women identified economic constraints, physical strain, and sedentary jobs as key barriers [34]. The lack of knowledge regarding the benefits of PA was another barrier identified, consistent with previous studies reporting insufficient recognition of the benefits of leisure-time activity [35]. Time constraints due to work-life balance were the most frequently cited, in line with numerous previous studies [36,37]. In addition, convenient lifestyles that reduce opportunities for outdoor activities were reported as barriers, suggesting that future health care apps should propose outdoor activities to address this issue.

Within sociocultural environmental factors, social networks emerged as a barrier, with concerns about colleagues’ perceptions making interruptions in sedentary behavior challenging. Participants requested discrete activity suggestions suitable for workplace environments via health care apps. Physical and environmental factors included natural and work environments. Further, weather conditions significantly influenced PA participation, with participants requesting activity suggestions suitable for indoor conditions during poor weather. Future health care apps should incorporate weather considerations into recommendations. Work environments, particularly teleworking facilities, were also significant barriers. Regarding this, the COVID-19 pandemic has altered lifestyles and increased concerns about inactivity. The 2023 National Health and Nutrition Survey [22] indicated that the average step counts have significantly decreased over the past decade, with teleworking likely contributing to reduced commuting-related activities. However, teleworking offers relative autonomy and fewer concerns regarding the perceptions of colleagues, potentially making interventions through health care apps more effective.

### Implications

Building on the findings above, users’ perspectives suggest that social and environmental contexts (eg, workplace norms and weather constraints) shape opportunities for PA and should be considered when designing personalized PA support in health care apps. For example, apps could provide low-burden, unobtrusive prompts to interrupt prolonged sitting in workplace settings (eg, suggesting brief, socially acceptable activities such as getting a drink, picking up a printout, or taking a short restroom break). In addition, when outdoor activity is less feasible because of weather conditions (eg, rain or extreme temperatures), apps could offer tailored indoor alternatives that align with users’ preferences and available space.

### Limitations

This study had some limitations. First, recruiting participants under the description “interviews regarding personalized exercise recommendation functions on health care applications” might have attracted participants already highly interested in exercise. Although 6 participants initially reported regular exercise habits, the interviews revealed that nearly all the participants were physically active, which may have biased the findings. Second, the sample (3 men and 8 women) likely skewed the results toward the perspectives of women. Considering the potential sex differences, future studies should ensure balanced sex representation. Third, the study was limited to users of a single health care app, which raises questions about its generalizability to other apps. Moreover, because CALOmama PLUS primarily focuses on diet and weight management, this context could have influenced how participants framed PA motivators and barriers. Therefore, our findings may be most applicable to digital interventions that embed PA promotion within diet or weight management apps, and themes may differ in primarily PA-focused or more general apps. Finally, this qualitative study was designed to elicit user perspectives and generate themes relevant to personalized PA support; it was not intended to elicit, rank, or prioritize specific app features.

### Conclusions

This FGI study explored users’ understanding of PA and identified multilevel motivators and barriers shaping their ability and willingness to be active. The findings highlight the importance of improving population-level communication of the PA concept and of considering users’ day-to-day contexts when designing personalized PA support. These user-informed insights can guide the future development of personalization approaches to PA promotion in health care apps.

## Supplementary material

10.2196/85390 Multimedia Appendix 1 Focus group interview guide.

10.2196/85390 Checklist 1 COREQ checklist.

## Abbreviations

COREQ: Consolidated Criteria for Reporting Qualitative Research
FGI: focus group interview
MVPA: moderate-to-vigorous physical activity
PA: physical activity
