# Supplementary material for: Understanding User Perspectives to Inform Personalized Physical Activity Promotion in a Health Care App: Qualitative Focus Group Interview Study
Source: JMIR Form Res. 2026 Mar 9;10:e85390. doi: 10.2196/85390 (PMC12978931; doi:10.2196/85390)
Supplement: Multimedia Appendix 1 [file formative-v10-e85390-s001.pdf]

Multimedia Appendix 2.

Focus group interview guide		
**Time***	**Stage**	**Contents**
13:00	Researcher's arrival at the study site	Set-up and discussion prior to implementation
13:30	Entry of eligible participants at the study site	
14:00 (5 mins)	Ice-breaking①	Greetings and introduction of the researchers
14:05 (5 mins)	Introduction of the study and informed consent process	
14:10 (5 mins)	Ice-breaking②	a. Explanation of the study objectives, procedures, and precautions<br>b. Self-introduction by participants
14:15 (10 mins)	Question 1	How do you perceive the importance of physical activity?
14:25 (10 mins)	Question 2	What are the motivating factors for engaging in physical activity?
14:35 (10 mins)	Break	
14:45 (10 mins)	Question 3	What are the barriers to engaging in physical activity?
14:55 (5 mins)	Summary and closing	Appreciation for the valuable feedback provided

* As an example of a single interview.
